# Immunotherapeutic targeting of calreticulin mutant myeloproliferative neoplasms

**DOI:** 10.1038/s41375-026-03115-w

**Published:** 2026-08-18

**Authors:** Charles Hertz, Nikolaos Spyrou, Ronald Hoffman, Cansu Cimen Bozkus, Nina Bhardwaj, Douglas Tremblay, Marina Kremyanskaya, John Mascarenhas

**Affiliations:** 1 SUNY Downstate Health Sciences University, Brooklyn, NY USA; 2 Icahn School of Medicine at Mount Sinai, New York, NY USA

**Keywords:** Myeloproliferative disease, Immunotherapy

## Abstract

*Calreticulin (CALR)* frameshift mutations drive the majority of *JAK2*/*MPL*-wild-type cases of essential thrombocythemia and myelofibrosis, producing a shared novel C-terminus that activates the thrombopoietin receptor and leads to constitutive Janus kinase (JAK)/signal transducer and activator of transcription (STAT) signaling. *CALR* mutations transform the multifunctional endoplasmic reticulum (ER)-resident protein into an oncogenic driver that aberrantly traffics to the cell surface, activates the thrombopoietin receptor, MPL, and promotes MF megakaryocytic proliferation and hemopoietic stem cell fitness. Clinically, *CALR*-mutated MPNs affect younger patients, exhibit thrombocytosis and progressive anemia, and, in MF patients, are associated with a superior survival compared with *JAK2*- or *MPL*-mutated disease, yet responses to hydroxyurea and ruxolitinib remain inferior. The C-terminal motif shared between CALR mutations is presented extracellularly on major histocompatibility complex (MHC) Class I molecules and in complex with the thrombopoietin receptor enabling targeting with vaccination and antibody based therapeutic platforms, respectively. Mutant CALR peptide vaccines induce T cell responses but have failed to result in hematologic or molecular responses. Mutant CALR-specific monoclonal antibodies, such as Fc-silent antagonists, can block mutant-CALR–MPL signaling, suppress hematopoietic stem and progenitor cells (HSPC) proliferation and megakaryopoiesis, and achieve rapid, durable hematologic remissions with minimal toxicity in early-phase trials. Preclinical antibody-drug conjugates, bispecific T-cell engagers, and chimeric antigen receptor T cell (CAR-T) cells also show potent, selective mutant-cell killing. Here we review the basis and ongoing translational and clinical efforts in the development of CALR-targeted immunotherapies that offer a potential shift from symptom management to disease-modifying treatment in this molecularly defined MPN subset.

## Introduction to myeloproliferative neoplasms and their genetic drivers

Polycythemia vera (PV), essential thrombocythemia (ET), and primary myelofibrosis (MF) comprise the three subtypes of *BCR-ABL1* negative myeloproliferative neoplasms (MPN). The diagnoses integrate clinical, morphologic, and molecular features outlined in the World Health Organization and International Consensus Criteria, reviewed elsewhere [1]. The common basis of these diseases consists of several well characterized driver mutations that affect hematopoietic cell signaling pathways leading to excessive production of mature myeloid cells.

MPNs are characterized by a constellation of symptoms including fatigue, pruritus, weight loss, and splenomegaly, depending on the subtype [2]. PV is the most prevalent MPN and is characterized by clonal erythrocytosis, often accompanied by leukocytosis, thrombocytosis, and increased risk of thrombotic complications. ET is the most indolent MPN, its main feature being isolated thrombocytosis. Both ET and PV are associated with increased risk of both arterial ischemic events (stroke, microvascular ischemia), ocular migraines, erythromelalgia, acquired von Willebrand’s disease and bleeding risk, and arterial or venous thrombosis. MF is the least prevalent, but most aggressive MPN and is associated with bone marrow fibrosis and eventual bone marrow failure, progressive splenomegaly, anemia, and variable changes in the platelet and leukocyte counts. A fraction of patients with chronic phase MPNs evolve to an aggressive form of secondary acute myeloid leukemia termed MPN blast phase which is associated with a dismal median overall survival of just 3-6 months [3].

In 2005, the identification of the *JAK2*^*V617F*^ mutation, transformed the understanding of these diseases [4]. One year later, *JAK2* exon 12 and *MPL* exon 10 mutations were discovered as the drivers of a small minority of cases [4]. In 2013, two landmark studies simultaneously identified somatic mutations in *CALR* (calreticulin) in the majority of *JAK2/MPL*-negative MPN patients [5, 6]. Triple-negative MPNs make up the other 5% of PMF patients and 12% of ET patients [7].

The acquired gain-of-function mutations in these three principal disease-driver genes (*JAK2*, *CALR*, and *MPL*), found in ~90% of MPNs, alone lead to constitutive activation of the JAK/STAT signaling pathway [8]. *JAK2*^*V617F*^ mutation uncouples cytokine binding from downstream growth signals in multiple myeloid growth pathways (EPO receptor, G-CSF receptor, and the thrombopoietin receptor-MPL), explaining its association with all three MPN subtypes. In contrast, *CALR* and *MPL* mutations specifically activate the thrombopoietin receptor MPL, restricting their occurrence to ET and PMF. Around 50-60% of patients harbor a sole mutation, but the rest harbor additional mutations affecting epigenetic regulator genes (e.g., *TET2*, *DNMT3A*, *IDH1/2*, *ASXL1*, *EZH2*), splicing genes (*SRSF2*, *SF3B1*, *U2AF1*), and signal transduction genes (e.g., *NRAS*, *KRAS*, *SH2B3*) [8]. The genomic landscape of MPNs is complex, with inter-patient variation in genetic and clonal architecture that influences phenotype and prognosis [2]. Management varies by disease subtype. In ET and PV, the primary goal is preventing thrombotic and hemorrhagic complications and monitoring for progression to MF or acute leukemia. In MF, treatment goals shift to controlling disease-related symptoms and splenomegaly, ameliorating cytopenias, and monitoring for progression to acute leukemia while also considering the potential curative role of allogeneic hematopoietic stem cell transplantation [2]. In patients with PV, current treatment focuses on reducing blood viscosity and preventing thrombosis with therapeutic phlebotomy, often supplemented by low-dose aspirin and cytoreductive agents like hydroxyurea or interferon-alpha. Cytoreduction with hydroxyurea, anagrelide, or pegylated interferon-alpha is the cornerstone of ET management in high-risk patients (older than 60 years, with history of thrombosis, or platelets exceeding 1500 × 10⁹/L). In patients with MF and constitutional symptoms, JAK2 inhibition (Food and Drug Administration (FDA)-approved options include ruxolitinib, fedratinib, pacritinib, and momelotinib) is the mainstay of treatment. Currently, allogeneic hematopoietic cell transplantation represents the only curative option and should be considered in appropriate candidates with MF who are at high risk of progression and compromised survival.

While JAK inhibitors have been found to play a role regardless of driver mutation, CALR-mutated disease offers an opportunity for mutation-specific therapeutics that target the mutant CALR neoantigen and take advantage of its unique cell surface expression. The remainder of this review will focus on *CALR-*mutated MPNs and the basis for targeted approaches with immunotherapeutics.

## Wild-type vs mutant CALR

### Wild-type CALR (wt-CALR)

**Fig. 1:**
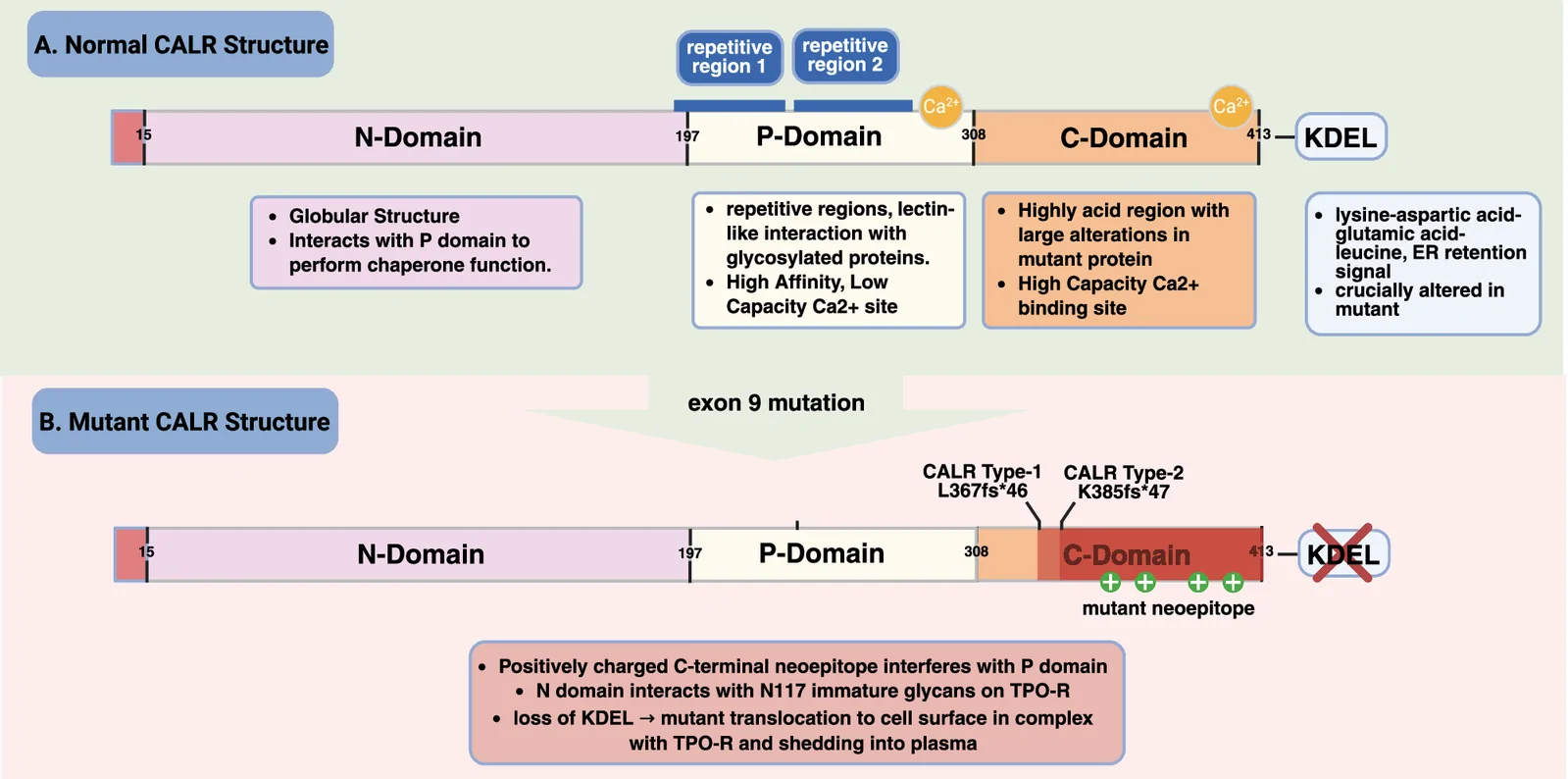
Structural organization of wild-type and mutant CALR and its role in oncogenic signaling. **A** Representation of wild-type CALR, highlighting the N-domain, P-domain, and C-domain, which together mediate chaperone activity, calcium binding, and endoplasmic reticulum (ER) retention via the C-terminal KDEL motif. The coordinated interaction between domains supports proper protein folding and intracellular trafficking. **B** Structure of mutant CALR resulting from exon 9 frameshift mutations (Type 1 and Type 2), leading to loss of the KDEL retention signal and generation of a positively charged C-terminal neoepitope. This altered C-terminus disrupts normal domain interactions, promotes aberrant trafficking to the cell surface, and enables binding to immature glycans on the thrombopoietin receptor (TPOR/MPL). The resulting mutant CALR–TPOR complex drives cytokine-independent receptor activation and constitutive JAK–STAT signaling in CALR-mutated myeloproliferative neoplasms. CALR, calreticulin; ER, endoplasmic reticulum; JAK–STAT, Janus kinase–signal transducer and activator of transcription; KDEL, lysine-aspartic acid-glutamic acid-leucine (endoplasmic reticulum retention signal sequence); TPOR/MPL, thrombopoietin receptor (myeloproliferative leukemia virus oncogene). Adapted from Lu et al. 2015 [16]. Created in BioRender. Hertz, C. (2026) https://BioRender.com/ywy4f53.

**Fig. 2:**
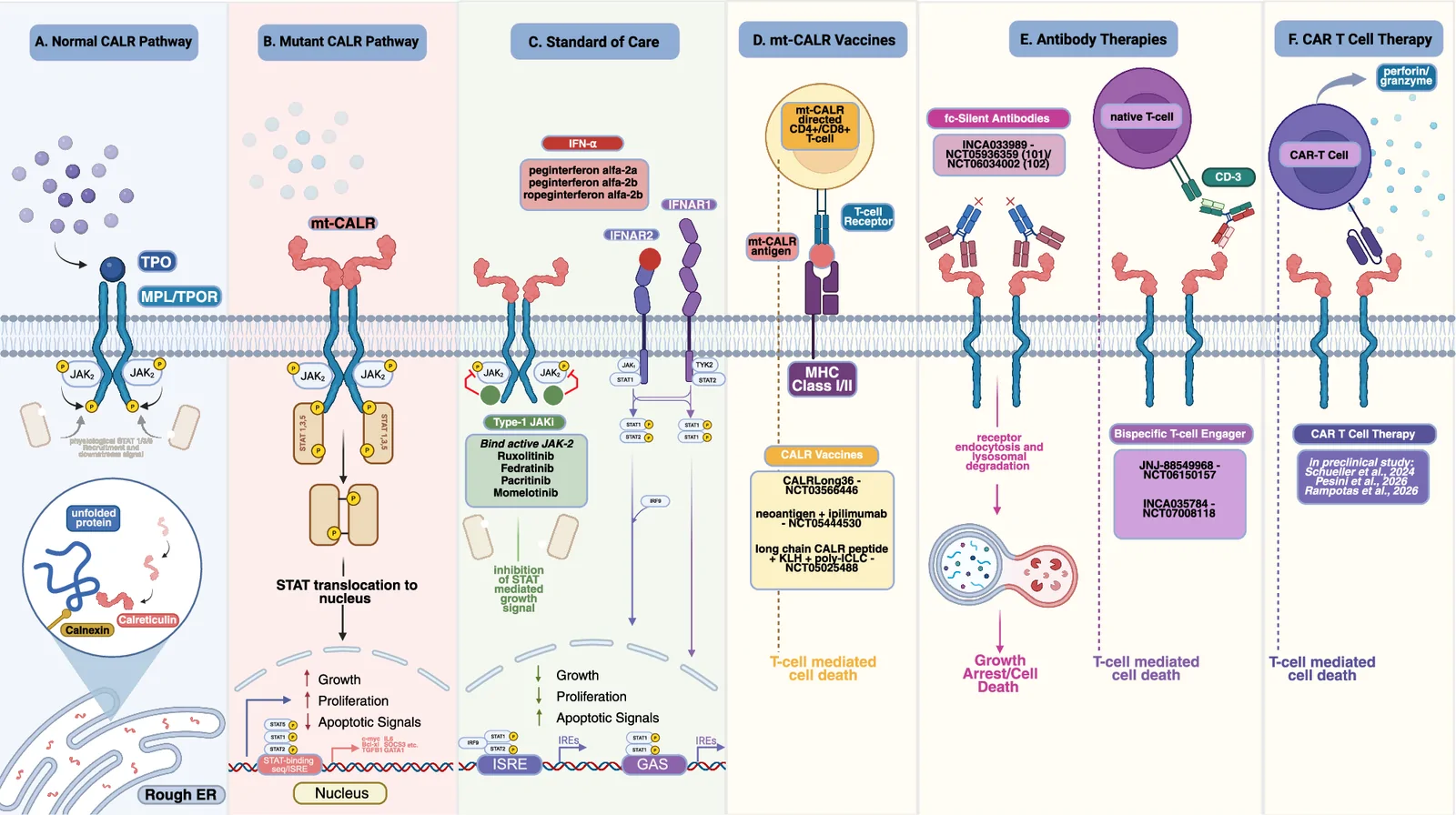
Mechanistic Illustration of Traditional and Novel Therapeutics in CALR-mutated MPNs. **A** Normal CALR pathway, illustrating physiologic thrombopoietin (TPO) binding to the thrombopoietin receptor (TPOR/MPL), resulting in regulated JAK–STAT activation and controlled hematopoietic proliferation and differentiation. Depiction of calreticulin and calnexin collaborative chaperone function in ER. **B** Mutant CALR pathway, demonstrating interaction between mutant CALR and MPL, leading to ligand-independent receptor activation, constitutive JAK–STAT signaling, and enhanced transcription of genes promoting proliferation and survival (c-Myc, Bcl-xL), cytokine signaling (IL6), fibrogenic signaling implicated in bone marrow fibrosis (TGF-β1), negative feedback regulation (SOCS3), and megakaryocytic/erythroid differentiation (GATA1), among others. **C** Standard of care, highlighting conventional therapeutic approaches including Type I JAK inhibitors (JAKi), which bind active JAK2, and interferon-alpha (IFN-α)-based therapies, which signal through IFNAR1/IFNAR2 to activate JAK1 and TYK2 and phosphorylate STAT1/STAT2; STAT1-STAT2-IRF9 complexes drive transcription via the ISRE, while STAT1 homodimers act through the GAS element, together comprising the interferon regulatory elements (IREs) depicted; these approaches primarily target downstream signaling and disease-related symptoms without directly inhibiting the mutant CALR–MPL complex. **D** Mutant-CALR vaccines (NCT03566446, NCT05444530, NCT05025488) illustrating neoantigen-targeted vaccination strategies designed to induce mt-CALR-specific T cell responses through antigen presentation on MHC Class I or II molecules, leading to CD4^+^/CD8^+^ T cell targeting of mutant hematopoietic cells. **E** Antibody-based therapies, including Fc-silent monoclonal antibodies (NCT06034002 (102)/ NCT05936359 (101)) and bispecific T-cell engagers (NCT06150157, NCT07008118), which selectively recognize mutant CALR on the cell surface to inhibit oncogenic signaling, or recruit T cells. **F** CAR-T cell therapy is not currently in the clinical trial phase. CARs recognize mt-CALR on the cell surface, inducing cytotoxicity. c-Myc, MYC proto-oncogene; Bcl-xL, B-cell lymphoma-extra large; CALR, calreticulin; CAR-T, chimeric antigen receptor T-cell; CD4 + /CD8 + , cluster of differentiation 4/8 (T-cell subset markers); GAS, gamma-activated sequence; GATA1, GATA binding protein 1; IFN-α, interferon alpha; IFNAR1/IFNAR2, interferon-alpha/beta receptor subunit 1/2; IL6, interleukin 6; IREs, interferon regulatory elements; IRF9, interferon regulatory factor 9; ISRE, interferon-stimulated response element; JAK1, Janus kinase 1; JAK–STAT, Janus kinase–signal transducer and activator of transcription; JAKi, JAK inhibitor; MHC, major histocompatibility complex; mt-CALR, mutant calreticulin; NCT, National Clinical Trial (ClinicalTrials.gov identifier); SOCS3, suppressor of cytokine signaling 3; TGFB1, transforming growth factor beta 1; TPO, thrombopoietin; TPOR/MPL, thrombopoietin receptor (myeloproliferative leukemia virus oncogene); TYK2, tyrosine kinase 2. Created in BioRender. Hertz, C. (2026) https://BioRender.com/ywy4f53.

*The Calreticulin* (*CALR*) gene is located on chromosome 19p13.2 and contains 9 exons spanning 4.2 kb of genomic DNA [9]. The protein is a 46 kDa calcium-binding lectin chaperone protein primarily localized to the ER lumen. The CALR protein is composed of three distinct domains: the N-terminal globular domain (N-domain), a centrally located proline-rich P-domain, and a terminal C-domain (Fig. 1). The N and P domains of CALR interact to perform its chaperone function. The P-domain also contains a high-affinity, low-capacity Ca^2+^ binding site (Fig. 1). The C-domain contains a low-affinity, high-capacity Ca^2+^ binding site and a terminal lysine-aspartic acid-glutamic acid-leucine (KDEL) sequence, a powerful ER retention signal (Fig. 1) [10, 11]. The CALR protein, alongside its partner calnexin, an integral membrane lectin chaperone interact with the Hsp70 family members to prevent the export of unfolded proteins and guide the proper refolding of glycoproteins (Fig. 2A) [12–14]. More than 50% of ER-calcium is associated with calreticulin proteins [15, 16].

The role of wt-CALR outside of the ER has been extensively reviewed elsewhere. In brief summary, *calreticulin* may play a role in normal hematopoiesis and exhibits distinct expression profiles in different myeloid lineages [17]. *Wt-CALR* also plays a role in calcium-dependent processes such as cell adhesion, integrin signaling, proper MHC-I antigen presentation, and immunologic cell death [18]. The dysregulation of *CALR* is an important feature of many malignancies, where it presents as a mixed prognostic factor depending on the specific diagnosis [18]. Finally, cell surface CALR plays an important immunologic role as a phagocytic, “eat-me”, signal in apoptotic and malignant cells in many human cancers, including acute myeloid leukemia (AML) [18]. CALR exists at the biological threshold between healthy cells and those deemed unsalvageable, maintaining cellular homeostasis, then acting as a potent apoptotic signal once the cells reach the point of no return, fated to undergo immunologic cell death [19].

### Mutant CALR

Mutations in *CALR* corrupt the chaperone function, transforming it into an aberrant constitutive activator of the JAK/STAT signaling pathway. All pathogenic *CALR* mutations occur in exon 9 and cause a + 1 base-pair frameshift, generating a shared 36 amino acid C-terminal neoantigen (Fig. 1) [5, 6]. Over 80% of these mutations fall into one of two categories: 53% are type-1 mutations, *CALR*^*del52*^ (L367fs*46), and 32% are type-2 mutations, *CALR*^*ins5*^ (K385fs*47) (Fig. 1) [15]. The remaining *CALR* mutation types are commonly categorized as type-1-like, type-2-like, or “other”. These mutations are almost always heterozygous and very rarely homozygous. Of the documented homozygous *CALR* mutations, all are 5 bp insertions [9]. These mutations share the development of a positively charged C-terminal neoantigen and the loss of the KDEL ER retention signal (Fig. 1) [6, 20]. The presence of positively charged residues in the C-terminus of the CALR protein induces a conformational change, where the mutant C-terminus interferes with the CALR P-domain (Fig. 1). Ultimately, this leads to an interaction between the CALR N-domain and immature glycans on the N117 residue of the thrombopoietin receptor (TpoR/MPL), hijacking the lectin function exhibited by normal CALR chaperones. Loss of the KDEL retention signal allows mobilization of mt-CALR out of the ER and subsequent shedding of mutated protein into the plasma and translocation of mt-CALR/MPL complexes to the cell surface (Fig. 1). Consequently, there is thrombopoietin (TPO)-independent MPL homodimerization, leading to constitutive JAK/STAT activation and accompanying downstream effects (Fig. 2B) [21–24]. In the presence of a *CALR* mutation, JAK2, ERK1/2 and STAT5 phosphorylation levels match those of TPO-exposed wt-CALR cells, indicating the uncoupling of cytokine binding from pathway activation [25]. The proliferative potential of *mt-CALR* relies on the presence of JAK2 and MPL/TpoR and is diminished with deletion of either protein or the addition of ruxolitinib [25–29].

The shedding of mt-CALR protein into the plasma may prevent effective anti-tumor host responses [30]. Given its cell surface expression, the spontaneous generation of mt-CALR-directed antibodies would be expected, yet these have not been detected in patients. CALR-mutated patients secrete mt-CALR protein into the extracellular space, and PMF patients exhibit higher levels of circulating CALR than controls [31, 32]. The high level of circulating mutant protein may saturate mutant-specific antibodies and induce tolerance in T-cells, explaining the lack of a spontaneous anti-tumor antibody or cell-mediated response in patients harboring the mutation [30].

## Clinical and prognostic implications of CALR mutation

*CALR* mutations are generally associated with a more indolent disease course and improved outcomes in both ET and MF when compared to matched *JAK2* mutant patients. Patients with *mt-CALR*-driven disease tend to be younger in age and predominantly male, exhibit more extreme thrombocytosis, more anemia, and less leukocytosis [6, 33–35]. *CALR* mutations are associated with a decreased thrombosis risk in ET and MF patients [6]. The lower thrombotic risk has been attributed to lower hematocrit levels and white blood cell (WBC) counts and less platelet reactivity, generating a more modest prothrombotic milieu compared to *JAK2* mutant MPNs [6, 36]. *CALR* mutations are also associated with lower rates of progression to blast-phase disease. The 10-year progression incidence was found to be 9% for *CALR*-mutated patients compared to 19% for *JAK2*^*V617F*^, 17% for *MPL*, and 34% for triple-negative disease [37]. *CALR* mutations are also significantly associated with increased overall survival in MF, with studies showing a median OS of 18 years for *CALR*-mutated patients versus 9 years in *JAK2*^*V617F*^/*MPL* mutations and only 3 years for triple-negative disease [37]. *CALR* mutations do not appear to influence the rate of progression or survival in ET [38, 39]. *CALR*-mutated patients also have improved survival and decreased non-relapse mortality after allogeneic stem cell transplant in MF [40]. *CALR* mutation subtypes are associated with distinct ET and MF clinical phenotypes. Among *CALR* mutation subtypes in MF, type-1 mutations have been associated with improved survival compared to type-2 [41]. Although no survival difference has been observed in ET, type-2 *CALR* mutations are associated with more extreme thrombocytosis [41].

The presence of *mt-CALR* does not currently influence the management of ET or MF. However, *mt-CALR* ET patients demonstrate significantly inferior responses to hydroxyurea. Complete hematological response rates at 12 months are only 40% for *CALR*-mutated patients versus 67% for *JAK2*^*V617F*^ + patients, and the duration of response is shorter [42]. In MF, *mt-CALR* patients were shown to have lower symptom and spleen response rates to ruxolitinib than *JAK2-*mutated patients [43]. Consistent with this, a large real-world analysis of ruxolitinib-treated MF patients found no significant difference in overall survival between *CALR*- and *JAK2*-mutated transplant-eligible patients (5-year OS 63.7% vs. 69.1%, *p* = 0.17), in contrast to the survival advantage associated with *CALR* mutation status in cohorts followed from diagnosis. The authors attribute this to *CALR*-mutated patients starting ruxolitinib with more severe baseline disease and after a substantially longer delay from the time of diagnosis (median 2.6 vs. 0.7 years), raising the possibility that the natural survival advantage of *CALR*-mutated disease is eroded by the time patients reach JAK inhibition, highlighting the role for early CALR-directed interventions [43].

Response to interferon is similarly mutation-dependent. Genomic profiling from the randomized DALIAH trial showed that interferon-alpha induces distinct molecular response kinetics in *CALR*-mutated compared with *JAK2*-mutated MPNs [44]. In those harboring a *CALR* mutation, complete hematologic remissions could not be correlated with decreases in *CALR* variant allele frequency (VAF), as was seen in patients harboring a *JAK*2 mutation. Alongside inferior ruxolitinib responses, the lack of a stronger effect on mutation-harboring HSPCs that sets interferons apart from hydroxyurea in *JAK2*-mutated ET patients further emphasizes the necessity for novel therapies in *CALR*-mutated patients.

## Targeting mt-CALR in MPNs with immunotherapy

All documented *CALR* mutations share a common C-terminal motif presenting a convenient neoantigen for the development of therapeutics that display mutant specificity regardless of the specific mutation type. This uniformity across mutation types also helps explain why immunotherapeutic strategies may succeed here, where they have largely failed in AML and MDS, illustrating the unique vulnerability of mt-*CALR*-driven MPNs to immunotherapeutic targeting. Principle surface targets (e.g., CD33, CD123) in AML are broadly co-expressed on normal HSPCs, producing on-target, off-tumor myelotoxicity that constrains immunotherapy intensity, and the generally low mutation burden means most neoantigens are patient-private rather than shared across patients [45, 46]. By contrast, mt-CALR is a public neoantigen displayed only in complex with TPOR, the expression of which is comparatively restricted to the megakaryocyte-erythroid/HSPC/eosinophil-basophil-mast cell compartment, affording both a broadly applicable target and a therapeutic window largely absent in AML [47–49]. Moreover, *CALR* is an MPN driver mutation rather than a bystander, rendering it a key target for cancer survival, making it harder for cancer cells to survive through clonal evolution. Furthermore, the indolent MPN course allows access to immunotherapy with a lower marrow blast burden and without the effects of T-cell-damaging cytotoxic chemotherapy frequently encountered in AML [50–52]. This uniquely targetable, MPN-driving, mutant CALR protein neoantigen is expressed extracellularly in complex with TpoR/MPL and presented by MHC Class I, posing an attractive target for vaccination, monoclonal antibody, and CAR-T therapeutic platforms, some of which (vaccines, bispecifics, and CAR-T cells) rely on the recruitment of functional T cell compartments lacking in AML [53, 54].

### Vaccines (m-CALR Vaccine)

Therapeutic cancer vaccines are emerging as a clinically impactful modality, with recent trials demonstrating that vaccination can improve patient outcomes [55]. In 2016, Holmstrom et al. documented spontaneous T cell responses to mutant CALR [56]. They followed up in 2018, demonstrating that T cells specific to the C-terminus of mutant CALR recognized autologous *mt-CALR* myeloid cells in the periphery and hematopoietic stem cells (HSCs) and induced cytotoxicity against HSCs [57]. Although spontaneous mt-CALR responses exist in patients, the response is rather weak, and vaccination can boost preexisting immunity as well as generate de novo immunologic response [50, 54]. Several trials (NCT03566446, NCT05444530, NCT05025488) have used long peptide vaccine modalities to target the C-terminal neoantigen general to all *CALR* mutation types (Table 1 and Fig. 2D*)*.

**Table 1:** Clinical trials of CALR-directed immunotherapies.

NCT No.	Intervention/Agent	Platform	Phase/Accrual/ Status/Key outcomes
NCT03566446	CALRLong36 long-peptide + Montanide Agent Specifications: Synthetic 36-aa peptide spanning the full mutant C-terminal frameshift sequence (RMRRMRRTRRKMRR KMSPARPRTSCREACLQGWTEA), shared across all CALR mutation types [30]	Vaccine	Safe; accrual, 10 patients; CALR-specific T cell responses (8/10 pts); no hematologic or molecular response [58, 59].
NCT05444530	VAC85135 + ipilimumab Agent Specifications: neoantigen regimen for CALR/JAK2V617F-mutant MPN patients, neoantigen epitope details not publicly available	Vaccine	Phase I completed; 14 patients enrolled; 36% mounted a detectable immune response but no clinical benefit was observed (blood counts, marrow fibrosis, or CALR/JAK2 VAF); further development discontinued [60].
NCT05025488	CALR long-peptide + KLH/poly-ICLC Agent Specifications: a pool of 6 overlapping synthetic long peptides (administered as two mixtures of three), spanning the 44-aa mutant CALR C-terminus [83]	Vaccine	Phase I recruiting; accrual goal, 10 patients; designed to boost antigen presentation and T cell priming.
NCT06034002 (102)/ NCT05936359 (101)	INCA033989 (Fc-silent anti-mt-CALR) Agent Specifications: Fully human, high-affinity, Fc-silenced monoclonal antibody targeting the C-terminal neoepitope in TPOR-complexed conformation; neoantigen epitope details not publicly available [65]	Antibody	**ET cohort (INCA033989-101/102)**: ET cohort as of Jan 2026; 110 enrolled, 106 continuing, well tolerated; 68% CHR, 17% PHR, median CHR duration 15 weeks (median onset 2.1 weeks); VAF reduction ≥25% correlated with durable CHR (*P* < 0.0001) and with megakaryocyte declustering (*P* = 0.02); marked type-1 vs non-type-1 disparity in hematologic response at C7D1 (100% vs 47% achieving platelets <600 G/L; 92% vs 33% achieving <400 G/L); supports FDA Breakthrough Therapy designation (granted Nov 2025) and planned Phase 3 ET trial (mid-2026) [67]. **MF monotherapy:** MF monotherapy as of Jan 2026; 70 enrolled, 84% continuing, no DLTs; 44% SVR25 (31% SVR35) at 24 weeks; TSS50 in 37%; anemia response in 58% (19 major responses); 91% achieved mt-CALR VAF reduction [68]. **MF + ruxolitinib combination:** MF-ruxolitinib combination cohort; 20 enrolled; 63% SVR25 (6/16 SVR35) at 24 weeks; TSS50 in 31%; VAF reduction in 95% of patients [68]. **Companion translational analysis:** NGS/single-cell translational analysis of 76 MF patients; clonal complexity (co-occurring mutations, including ASXL1) did not significantly affect spleen or anemia response rates; 92% achieved VAF reduction by NGS; single-cell analysis showed HSPC reduction in all SVR25 responders [69].
NCT06150157	JNJ-88549968 (anti-mt-CALR × CD3) Agent Specifications: Bispecific T-cell redirecting antibody targeting mt-CALR epitopes common to all CALR mutation types, paired with CD3 on T cells [70].	BiTE	Phase I recruiting; accrual goal, 100 patients, pre-clinical data show selective binding and xenograft efficacy [70].
NCT07008118	INCA035784 (anti-mt-CALR × CD3) Agent Specifications: Bispecific T-cell-redirecting antibody targeting a conserved N-domain epitope on surface-exposed mt-CALR common to both type 1 and type 2 mutations [71].	BiTE	Phase I recruiting; accrual goal: 120 patients. Preclinical data show selective binding and minimal CRS-associated cytokine release [71].

*ASXL1* ASXL1 gene, *BiTE* bispecific T-cell engager, *CALR* calreticulin, *CD3* cluster of differentiation 3, *CHR* complete hematologic remission, *DLT* dose-limiting toxicity, *FDA* Food and Drug Administration, *HSPC* hematopoietic stem/progenitor cell, *KLH* keyhole limpet hemocyanin, *mt-CALR* mutant calreticulin, *NCT* National Clinical Trial (ClinicalTrials.gov identifier), *NGS* next-generation sequencing, *PHR* partial hematologic remission, *poly-ICLC* polyinosinic-polycytidylic acid stabilized with poly-L-lysine and carboxymethylcellulose, *SVR25/SVR35* ≥25%/ ≥ 35% spleen volume reduction, *TPOR* thrombopoietin receptor, *TSS50* ≥50% reduction in total symptom score, *VAF* variant allele frequency.

The first vaccine trial, NCT03566446, tested a 36-amino-acid peptide, CALRLong36, targeting the mutant C-terminus of the CALR protein with Montanide (SEPPIC, Paris, France) as adjuvant (Table 1) [30]. Fifteen vaccines were administered to 10 patients over the course of one year and were determined to be safe and tolerable. The clinical trial showed evidence of immune activity, with the vaccine inducing T cell responses to the CALRLong36 vaccination peptide in 8/10 patients tested (4 patients had preexisting immunity, which was boosted post-vaccination, and 4 patients developed immunity after vaccination). These findings did not correlate with any clinical or molecular response. Further studies of marrow aspirates from the CALRLong36 study showed that vaccine administration failed to enrich the bone marrow T cell receptor (TCR) repertoire with mt-CALR-specific T cells [58]. The researchers determined that mutant-specific T cells were vastly outnumbered by their malignant targets, with an estimated time to disease control of hundreds to thousands of days and a maximum controllable variant allele frequency (VAF) lower than 1% [58]. Accordingly, Holmstrom et al. postulated two potential challenges to the development of vaccination therapies: generating an effective immune response within the immunosuppressive, chronically inflamed tumor microenvironment typical of MPNs and achieving bone marrow enrichment by mutant-directed T cells in the presence of an overwhelming population of circulating mt-*CALR* myeloid cells [58, 59]. Additionally, immunophenotypic analysis of mt-CALR-directed T cell subsets revealed that more patients had a CD4 response than a CD8 response, which is the T cell population exhibiting direct cytotoxicity against cancer cells [30]. Although CD4+ cells are critical and can display cytotoxic potential themselves, their effect is usually mediated by the robust activation, sustained expansion, and long-term memory of neoantigen-specific CD8+ effectors. Further potential explanations for the lack of clinical impact include low-affinity human leukocyte antigen (HLA) subtypes, defects in the MHC-mediated presentation of calreticulin neoepitopes, or poor immune stimulation by the adjuvant [53].

In NCT05444530, a neoantigen vaccine concurrently with ipilimumab, a monoclonal antibody that functions as a CTLA-4 antagonist, was administered, and in NCT05025488, a long peptide vaccine was given with keyhole limpet hemocyanin (KLH) and poly-ICLC (Hiltonol; Oncovir, Washington, DC, USA), to increase the effective immune response to administered vaccination targets (Table 1). Preliminary results from NCT05444530 were reported at EHA 2025: although 36% of patients receiving VAC85135 plus ipilimumab mounted a detectable immune response, no clinical benefit was observed, and further development of VAC85135 has since been discontinued [60]. The results of NCT05025488 are eagerly awaited to determine whether the challenges of NCT03566446 can be overcome by optimizing and broadening the T cell response.

### Antibodies

#### Preclinical studies

The mt-CALR surface neo-epitope can be targeted with antibodies. In 2021, Kihara et al. developed B3, the first antibody capable of recognizing mt-CALR in mice and donor cells from ET and PMF patients [61]. A mouse chimeric antibody of B3 was generated and administered for 5 days, showing a marked decrease in thrombocytosis in *mt-CALR-type-1* mice [61]. In 2021, Achyutuni et al. developed an IgG2a antibody against the human CALR oncoprotein and administered this to *mt-CALR* mice [62]. They observed the specificity and rapid attenuating effect on thrombocytosis exhibited by the B3 antibody. This study also revealed that solubilized mt-CALR did not provide a significant antibody sink or diminish the potency of thrombocytosis-attenuating effects [62]. In 2022, Mughal et al. immunized mice using CALR C-terminal peptides to develop eight murine antibodies [63]. Analysis of these antibodies identified essential contact residues with the mt-CALR C-terminal neoepitope but did not confirm whether the antibody was capable of binding to mutant CALR dimers already complexing with MPL [63]. That same year, Tvorogov et al. provided this confirmation using an IgG2a rat antibody (4D7) [64]. 4D7 was shown to disrupt the binding of mt-CALR dimers to MPL, blocking signal transduction and extending survival in both *CALR* type-1 and type-2 xenografted murine models. Further, the disruption of this signaling also inhibited differentiation of myeloid progenitors into megakaryocyte lineages [64]. While these antibodies were developed in nonhuman species, these studies provided proof of principle for generating mt-CALR-specific antibodies and hinted at their potential clinical efficacy. Based on these studies, a variety of highly specific antibody-based therapeutic platforms have been developed, including Fc-silent antibody antagonists, antibody-drug conjugates, and bispecific T-cell engagers (BiTEs).

### Fc-silent antibody antagonists

INCA033989 is a first-of-its-kind human-derived IgG1 antibody antagonist of mt-CALR oncogenic function that, in preclinical testing, appeared mutant-specific, potently antagonistic, potentially disease-modifying, and reasonably safe (Fig. 2E) [65]. INCA033989 has specificity to mt-CALR in complex with MPL (TpoR) with a slightly higher affinity and slower dissociation in type-1 cells when compared with type-2. Both ex vivo and in vivo analyses showed that INCA033989 is a potent antagonist of STAT5 signaling and subsequent cellular proliferation. Ex vivo, INCA033989 administration resulted in broad inhibition of cytokine-independent cell proliferation (inhibition of pSTAT5, pSTAT3, pERK, pMEK, pAKT, pmTOR) in *Ba/F3* and *UT-7* cell lines and an attenuating effect on cell survival of *Ba/F3-TpoR* cells harboring a type-1 *CALR* mutation. In mouse surrogates that underwent competitive transplantation with type-1 mutant *CALR* cells, treatment with INCA033989 prevented aberrant thrombocytosis with no evidence of myelosuppression. The antibody also exhibited stronger effects on marrow morphology in transplanted mice with inhibition of mutant CD34 + HSPC proliferation, normalizing histology and the number of megakaryocytes. Finally, the antibody exhibits an Fc region mutation, silencing effector functions and reducing potential adverse effects related to host immune cell recruitment or complement activation, such as antibody-mediated cytotoxicity [65, 66].

Clinical data presented at EHA 2026 in ET patients participating in the INCA033989-101 (NCT05936359, Table 1), excluding the US study, and INCA033989-102 (NCT06034002, Table 1), indicated tolerability and efficacy [67]. One hundred ten ET patients were enrolled at doses ranging from 24 to 2500 mg intravenous infusion given every two weeks, with a median exposure of 28 weeks (14 doses). Concomitant use of hydroxyurea or anagrelide was allowed at the start of therapy. One hundred six patients remain in the study. The most common adverse events observed were fatigue (24%) and headache (20%), with 18 patients experiencing Grade 3 or greater treatment-emergent adverse events (TEAE).

Rapid normalization of hematologic parameters was seen across all dose levels. Partial hematologic remission (PHR) was defined as platelets ≤ 600 × 10^9^/L, leukocytes < 10 × 10^9^/L and complete hematologic remission (CHR) was defined as platelets ≤ 400 × 10^9^/L, leukocytes < 10 × 10^9^/L. 68% of patients met criteria for CHR, and an additional 17% met criteria for PHR [67]. Durable CHR and PHR were defined as meeting hematologic remission criteria for ≥ 12 weeks. The median duration of CHR was 15 weeks, with a median time to onset of 2.1 weeks. Notably, at C7D1 in patients exposed to ≥ 750 mg doses, 100% of type-1 vs 47% of non-type-1 patients achieved platelets <600 ×10^9^/L and 92% of type-1 vs 33% of non-type-1 patients achieved platelets <400 ×10^9^/LL indicating a disparity in hematologic response rates when comparing type-1 and non-type-1 mutated patients. VAF reductions ≥ 25% correlated with durable CHR (*P* < 0.0001). Among 38 patients who achieved CHR and ≥ 1 post-baseline VAF assessment, 71% achieved a ≥ 25% reduction in *CALR* mutant VAF. Histopathologic analysis showed a reduction in megakaryocyte clustering from baseline to C7D1, correlating with a ≥ 25% reduction in VAF (*P* = 0.02). This data supports the FDA Breakthrough Therapy designation granted in November 2025 and the planned initiation of a Phase 3 ET trial in mid-2026.

Clinical data presented at EHA 2026 for MF patients exposed to INCA033989 as a single agent and in combination with ruxolitinib reflected the tolerability seen in ET patients and showed indications of efficacy [68]. As of Jan 2026, 70 patients were treated with INCA033989 as a single agent, with 84% remaining in the study. Of enrolled patients, 59% (41) exhibited type-1, 21% (15) exhibited type-2, and 20% (14) exhibited type-other *CALR* mutations. The median exposure was 40 weeks (20 doses), with no dose-limiting toxicities (DLT) observed, and the maximum tolerable dose was not reached. Sixty-six patients had TEAEs. Twenty-one experienced TEAEs ≥ grade 3, most commonly anemia (9%) and neutropenia (7%).

Reductions in spleen volume, anemia responses, and decreased symptom scores, set alongside VAF and *mt-CALR* HSPC reductions, were signals of clinical efficacy and potential disease modification [68]. At 24 weeks, 44% (20/45), patients achieved spleen volume reductions of ≥ 25% (SVR25), including 31% (14/45) who achieved a reduction of ≥ 35% (SVR35). Symptom scores decreased by 50% (TSS50) in 37% (14/38) of evaluable patients. Of 36 patients with anemia and ≥ 12 weeks of treatment exposure, anemia responses were observed in 58% of patients (21), with 19 major responses defined as ≥ 12 weeks transfusion-free in baseline transfusion-dependent patients and a ≥ 1.5 g/dL hemoglobin increase averaged over 12 weeks, and 2 minor responses defined as a ≥ 50% reduction in transfusion requirements over 12 weeks. 91% (51/56) of patients in the study achieved a VAF reduction. In most patients, exploratory single-cell analysis showed a reduction in mt-CALR CD34+ cells, confirming the on-target effect of the antibody.

A translational analysis of 76 MF patients treated with INCA033989 was also presented at EHA 2026 using next-generation sequencing (NGS), and single-cell sequencing revealing that clonal complexity did not impact rates of spleen volume reduction or anemia response and demonstrated rapid reductions of mt*-CALR* HSPCs supporting the disease modifying activity of INCA033989 [69]. Among patients with co-occurring mutations and spleen assessments, SVR25 was achieved in 56% (35/63), including 40% (25/63) who achieved SVR35. This did not differ significantly from values in patients regardless of co-occurring mutation, where SVR25 was achieved in 57%, including 42% who achieved SVR35. Anemia response was achieved in 51% (23/45) of all patients, 54% (21/39) of patients with a co-occurring mutation, and 46% of patients with an ASXL1 mutation. Reductions in mt-*CALR* VAF via NGS occurred in 92% of patients, with 7 achieving a ≥ 25% reduction. Single-cell sequencing identified a reduction in HSPCs in all patients with SVR25.

In the combination cohort, 20 patients were treated with INCA033989 added to their stable dose of ruxolitinib [68]. All patients had TEAEs; 13 had ≥ grade 3 TEAEs, most commonly anemia (35%). At 24 weeks, 63% (10/16) of patients achieved SVR25, including 6 who achieved SVR35. 31% (4/13) achieved TSS50. Of 16 patients with anemia, 13% (2) achieved a major anemia response and 19% (3) had a minor response. VAF reduction occurred in 18 patients (95%).

Taken together, the EHA 2026 clinical dataset across both ET and MF cohorts substantiates INCA033989’s potential to modify *CALR*-mutated MPN biology, with high rates of hematologic and molecular response, VAF reductions that track closely with response depth, and a companion NGS/single-cell analysis confirming that clonal complexity does not blunt clinical benefit [67–69]. This efficacy has been achieved without the myelosuppressive toxicity characteristic of ruxolitinib, hydroxyurea, and interferon. Further data are eagerly awaited to determine whether INCA033989 at higher doses demonstrates comparable efficacy in MF and ET patients harboring non-type-1 disease and ultimately if patients can achieve deep molecular responses over time.

### Bispecific T-cell engagers (BiTEs)

As with INCA033989, the CALR neoepitope provides an attractive tumor-specific antigen for targeting with BiTEs. JNJ-88549968 (NCT06150157) and INCA035784 (NCT07008118) are two currently active studies using BiTEs for the treatment of MPNs (Table 1) (Fig. 2E).

JNJ-88549968, currently in phase I testing, is a first-of-its-kind bispecific T-cell engager targeting *CALR-*mutated MPN cells. Preclinical testing shows that JNJ-88549968 selectively binds *CALR*-mutated cells without binding to wild-type cells [70]. Initial indications of efficacy and specificity were seen with induced cytotoxicity in mt-CALR CD34+ cells regardless of CALR mutation type. In vivo efficacy of JNJ-88549968 was also demonstrated by significantly increasing the lifespan of a treated xenograft murine model. Importantly, soluble CALR protein had no evaluable impact on JNJ-88549968 activity.

INCA-035784, a second T cell redirecting antibody in early clinical development, takes a mechanistically distinct approach, targeting the conserved N-domain of surface-exposed mt-CALR shared across type 1 and type 2 mutations rather than the C-terminal neoepitope [71]. INCA-035784 selectively bound both mutation types without engaging wild-type CALR, activated and expanded CD8 + T cells, and induced T-cell-mediated cytotoxicity against both engineered and primary patient-derived mt-CALR + CD34+ cells without triggering non-specific cytokine release. In an autologous xenograft model derived from a myelofibrosis patient with a high mutant allele burden, INCA035784 reduced malignant myeloid and megakaryocytic populations while sparing erythroid progenitors. Encouragingly, INCA-035784 induced minimal levels of CRS-associated cytokines (not quantified in abstract); promising evidence for a more favorable side-effect profile when compared to other BiTEs such as mosunetuzumab (approved for follicular lymphoma) and vibecotamab (currently being studied in AML and MDS), targeting CD20 and CD123, respectively.

More recently, Nishimura et al. employed a therapeutic strategy like that of INCA035784, targeting non-mutated regions of the extracellularly expressed mt-CALR protein. This bispecific induced cytotoxicity against CALR-mutated cells in preclinical models, lending additional preclinical evidence for the strategy employed in INCA035784 [72]. Finally and importantly, murine bispecific antibody DX1-2C11, targeting the mutant C-terminal linear epitope, activated CD4+ and CD8 + T cells within 24 hours and durably depleted mt-CALR-expressing HSPCs in vivo, providing encouraging preclinical evidence that T cell-redirecting therapy may achieve a favorable therapeutic index in *CALR*-mutated MPNs that have proven challenging to establish in AML [73].

While some encouraging preliminary safety data exist for INCA-035784, T cell involvement introduces potential side effects such as cytokine release syndrome (CRS) and immune effector cell-associated neurotoxicity syndrome (ICANS), which are avoided by an Fc-silent antibody such as INCA033989. While the safety and efficacy data from these studies are eagerly awaited, safety data from other BiTEs in hematologic malignancies would draw caution and prompt careful patient selection to balance potential immune toxicity with efficacy.

### Antibody-drug conjugates

Conjugation of cytotoxic payloads to mutant-directed antibodies is another potential therapeutic approach. Fultang et al. presented preclinical data on monoclonal antibodies conjugated to SWI/SNF-related matrix-associated actin-dependent regulator of chromatin subfamily A members 2/4 (SMARCA2/4) dual degraders and cyclin-dependent kinase 9 (CDK9) degraders. As with previously mentioned *mt-CALR*-specific antibodies, the precision antibody-drug conjugate (pADC) exhibited selective and high-affinity binding to mutant cells [74]. Rapid internalizing of drug conjugates and selective degradation of molecular targets led to cytotoxicity in mt-CALR cells. Both SMARCA2/4 and CDK9-degrading pADCs exhibited robust inhibition of megakaryocytic and platelet differentiation, depleting cultured *mt-CALR* CD34+ cells, while sparing healthy cells. The evaluation of precision antibody-drug conjugates in humans is awaited.

### CAR-T (Fig. 2F)

In 2024, Schueller et al. murine studies demonstrated mt-CALR-directed CAR-T cells (CAR-T) to be a highly mutation-specific and effective immunotherapeutic strategy for reducing leukemic burden in *CALR*-mutated disease [75]. Schueller et al. showed that CAR-T cells generated via retroviral transduction of CD3/CD28-activated splenic T cells effectively eliminated Ba/F3 cells expressing a type-1 *CALR* mutation and TpoR [75]. Comparable efficacy was observed in vivo, with transplanted NOD-sid gama (NSG) mice demonstrating complete tumor clearance between days 25 and 63 of treatment. Specificity was confirmed by co-culture experiments, in which mutant cells were eliminated while non-mutated cell lines were spared [75]. Notably, infusion of CAR-T cells in immunocompetent mice did not show a decrease in mutant cells, which the authors attributed to the potential “sink” effect of soluble mt-CALR protein.

In 2026, Pesini et al. expanded the preclinical understanding of mt-CALR-directed CARs by identifying anti-apoptotic signaling dysregulation as a mechanism of therapy resistance, thereby postulating the potential for synergistic treatment with venetoclax [76]. Mt-CALR CAR-Ts demonstrated mutant selectivity and effective leukemic cell clearance in xenografts. Transcriptomic profiling of residual malignant cells in mouse spleens revealed up-regulation of anti-apoptotic Bcl-2, the expression of which diminished CAR-T efficacy. Co-treatment with venetoclax restored efficacy to levels commensurate with transcriptomically normal cells, revealing yet another potentially synergistic pathway whereby CAR-T might be incorporated alongside standard chemotherapeutic agents. Encouragingly, no reduction in the cytotoxic effect of CAR-T cells was seen in samples enriched with soluble mt-CALR protein.

Later in 2026, Rampotas et al. furthered the preclinical basis for CAR-T cell therapy in MPNs using human “chimeroid” models to mimic the complex tumor microenvironment typical of MPNs and exhibited effective CAR-T cell clearance of CALR mutant cells in this setting [49]. Rampotas et al. also modeled a variety of relevant scenarios, further contextualizing the potential for CAR-T cells within the existing therapeutic landscape of MPNs. In patients treated with ruxolitinib and momelotinib, no decrease in CAR-T effectiveness was observed. Eltrombopag boosted the expression of TPO-R and, subsequently, mt-CALR in leukemic blasts and was correlated with increased CAR-T cell-mediated killing [49]. Cocultures of mt-CALR and wild-type cells were prepared to test the efficacy of CAR-T across a range of simulated VAFs. When present at 10%, mt-*CALR* CAR-T cells depleted 96.7% of cells harboring the mutation. Additionally, quantification of VAF reduction in type-1 and type-2 mutated cells, including samples harboring high molecular risk mutations (EZH2, ASXL1, CSF3R), showed decreases from 20 to 79% in response to CAR-T coculture. Last, long-term cytopenias following CAR-T have been associated with inflammatory damage of the hematopoietic niche. Increases in inflammatory genes associated with IFN-γ were observed in organoids engrafted with CAR-T cells, indicating an inflammatory response in bystander cells, including non-malignant populations.

In the absence of randomized comparisons between cellular therapies and antibody approaches, immunotherapeutic selection will likely be guided by physician- and patient-level assessments of cost, benefit, and the clinical scenarios in which each modality is most appropriate. These agents can be postulated to differ along three central axes: depth of response, toxicity, and accessibility. INCA033989 is administered serially to suppress mt-CALR-driven hematopoiesis, and over time, exposure to antibody antagonism appears to decrease malignant HSC burden, but effective clonal eradication has not yet been shown. Comparatively, CAR-T offers the potential for single-course clonal eradication with a more direct and intuitive cytotoxic effect. Preclinical modeling by Schueller et al. and Pesini et al. presents conflicting findings regarding the potential impact of soluble mt-CALR protein on CAR-T efficacy. Special interest will need to be paid in future preclinical and clinical studies to further describe this interaction and its impact on CAR-T efficacy.

The depth of clonal eradication must also be weighed against the potential for cytotoxicity. The involvement of T cells introduces potential life-threatening adverse reactions such as CRS/ICANS. Further, as the mt-CALR TPO-R complex is expressed on HSPCs, the potential for severe myeloablative effects, especially in patients with high clonal burden, as is often the case in MF and ET patients with advanced disease, cannot be ignored. This risk is only partially mitigated by the preclinical data described above. Across samples spanning mutant clonal burdens, CAR-T selectively depleted the mutant clone without cytotoxicity towards co-existing wild-type HSCs, with even the highest-burden samples retaining a residual non-mutant population [49]. The authors note that whether normal HSCs can adequately restore hematopoiesis after CAR-T therapy in myeloid malignancies remains unclear—leaving open whether a favorable treatment window exists for the application of CAR-T therapeutics in patients with advanced disease exhibiting near-total clonal replacement and minimal residual normal hematopoiesis [49]. Against the notably clean risk profile of INCA033989, which has exhibited minimal myelosuppression thus far, the toxicity of CAR-T cell therapy may be a significant differentiating factor both in physician decision-making and in patient comfort when approaching therapy. The broad application of CALR-directed CAR-T cell therapy may also be limited by accessibility: the manufacturing cost of these therapies drives costs per patient into the hundreds of thousands and even millions of dollars [77]. CAR-T persistence itself carries a similar trade-off: longer persistence has been associated with improved relapse-free outcomes but also with a higher incidence of delayed immune toxicity, including ICANS [78]. Consistent with this, cytopenias extending beyond the acute lymphodepletion period ( > 30 days post-infusion) have been linked to CAR T product characteristics that govern persistence as well as CRS/ICANS, often necessitating transfusion support, growth-factor administration, or thrombopoietin receptor agonists—an additional burden of care that compounds the cost considerations raised above [79]. Notably, bispecifics may sit somewhere in the middle ground as off-the-shelf, scalable T cell recruiting agents with lower manufacturing costs but with potentially less depth of response and toxicity than CAR-T. Additional preclinical and clinical data will help to further this debate and define which therapeutic platform balances these considerations most effectively.

## Conclusion

The discovery of CALR exon 9 mutations has reshaped the biological and therapeutic landscape of MPNs, establishing mt-CALR as a uniquely targetable neoantigen. The therapeutic developments that have taken place since may signal a shift in MPN therapy away from JAK-STAT inhibition and symptom management towards disease-modifying agents that may provide a curative alternative to stem cell transplantation. Although early vaccine strategies demonstrated immunogenicity without correlation with clinical benefit, the emergence of highly specific antibody-based platforms, including Fc-silent antagonists, antibody–drug conjugates, bispecific engagers, and CAR-T cells, may hold promise as disease-modifying agents in MPNs. Careful consideration of efficacy and tolerability in patients with low-risk disease, as is often the case in *CALR-*mutated MPNs, would be necessary in the evolving clinical investigation efforts. While INCA033989 shows promising preliminary safety and efficacy, complete data and final readout of ongoing clinical trials will clarify which strategy strikes this balance most effectively.

The evolution of therapeutic endpoints in ET and PV provides a framework for defining meaningful disease modification in *CALR*-mutated ET and MF (Table 2). The efficacy of mt-CALR-directed immunotherapies as disease-modifying agents will depend on their ability to achieve traditional outcomes alongside reductions in mt*-CALR* VAF, molecular remissions, clonal eradication, and restoration of normal polyclonal hematopoiesis. If mt-*CALR* VAF reduction is to serve as an indicator of on-target disease modification, the field must define what constitutes a clinically meaningful threshold. In J*AK2*^*V617F*^-mutated ET and PV treated with IFN or ruxolitinib, a ≥ 50% reduction in allele burden defines molecular response per European LeukemiaNet (ELN) criteria, and recent data suggest that a VAF reduction of ≥35% after 2 years predicts durable molecular response and reduced progression to myelofibrosis [80]. The PROUD and CONTI-PV trials established an “operational cure” threshold of J*AK2*^*V617F*^ VAF < 10% combined with sustained hematological response, a benchmark now used by French investigators to guide interferon discontinuation [81]. Whether analogous thresholds apply to mt-*CALR* disease remains undefined.

**Table 2:** Recounting evolving endpoints in MPNs and looking forward.

Therapeutic paradigm	Representative studies	Mechanism of action	Clinical endpoints	Molecular endpoints	Interpretation
Hydroxyurea in PV/ET	PVSG-08; CYTO-PV [84]	Non-specific cytoreduction of proliferating hematopoietic cells	Hematocrit control ( < 45%); platelet normalization; reduction in thrombosis	Not routinely assessed	Clinical benefit defined by hematological control without assessment of underlying clonal disease burden
Interferon in PV/ET	Quintás-Cardama et al [85].; MPN-RC 112; PROUD-PV/CONTINUATION-PV [81]	Immunomodulatory suppression of malignant hematopoietic clones	Complete hematological response; normalization of counts	Reduction in driver mutation allele burden (JAK2, CALR); partial and complete molecular responses	Introduction of molecular response as a measurable indicator of disease modification
JAK inhibition in MF	COMFORT-I; COMFORT-II [86]	Inhibition of JAK–STAT signaling and downstream inflammatory cytokine production	≥35% spleen volume reduction; ≥50% symptom improvement; overall survival (secondary)	Not routinely assessed	Clinical benefit reflects modulation of disease phenotype without direct targeting of the malignant clone
CALR-directed immunotherapy in MF (proposed)	Vaccines, Antibody-antagonists, bispecific engagers, CAR-T	Selective targeting of mutant CALR neoepitope on malignant hematopoietic cells	Spleen volume reduction; symptom improvement; hematological response	Reduction in CALR variant allele frequency; molecular response; depletion of mutant hematopoietic progenitors; improvement in bone marrow fibrosis	Maintains phenotypic measurement for comparison with JAK inhibitors while additional molecular end points align with potentially disease modifying drug mechanism

*CALR* calreticulin, *ET* essential thrombocythemia, *JAK* Janus kinase, *JAK2* Janus kinase 2, *MF* myelofibrosis, *PV* polycythemia vera.

PVSG-08, CYTO-PV, MPN-RC 112, PROUD-PV, CONTINUATION-PV, COMFORT-I, and COMFORT-II are established clinical trial names, not general abbreviations, and are not expanded here.

Although *CALR*-mutated MPNs generally carry a more favorable prognosis in the chronic phase, patients who progress to blast phase face uniformly dismal outcomes. Blast-phase MPN (MPN-BP) patients exhibit a dismal median overall survival of only 3–6 months, with 1- and 3-year survival estimates of <20% and <5%, respectively [3]. Even with contemporary therapies including hypomethylating agent (HMA)–venetoclax combinations, median survival remains approximately 10 months, and outcomes are not significantly improved without allogeneic stem cell transplantation [82]. Given the absence of effective standard therapies, this population represents an appropriate cohort for testing novel mt-*CALR*-directed immunotherapies, where the potential for disease modification may outweigh the risks of potential toxicities. Overall, mt-CALR-directed immunotherapies represent a potential paradigm shift towards personalized, disease-modifying care in MPNs, though the appropriate context of their application and definition of therapeutic success will need to be queried and defined to optimize their development and utilization.
